# Stem Cell Therapy in Treating Epilepsy

**DOI:** 10.3389/fnins.2022.934507

**Published:** 2022-06-27

**Authors:** Bao-Luen Chang, Kuo-Hsuan Chang

**Affiliations:** ^1^Department of Neurology, Chang Gung Memorial Hospital-Linkou Medical Center, Taoyuan City, Taiwan; ^2^School of Medicine, College of Medicine, Chang Gung University, Taoyuan City, Taiwan

**Keywords:** stem cells, seizure, epilepsy, disease-modifying, genetic engineering, graft, disease models

## Abstract

Epilepsy is a common disabling chronic neurological disorder characterized by an enduring propensity for the generation of seizures that result from abnormal hypersynchronous firing of neurons in the brain. Over 20–30% of epilepsy patients fail to achieve seizure control or soon become resistant to currently available therapies. Prolonged seizures or uncontrolled chronic seizures would give rise to neuronal damage or death, astrocyte activation, reactive oxygen species production, and mitochondrial dysfunction. Stem cell therapy is potentially a promising novel therapeutic strategy for epilepsy. The regenerative properties of stem cell-based treatment provide an attractive approach for long-term seizure control, particularly in drug-resistant epilepsy. Embryonic stem cells (ESCs), mesenchymal stem cells (MSCs), neural stem cells (NSCs), induced pluripotent stem cells (iPSCs), and adipose-derived regenerative cells (ADRCs) are capable of differentiating into specialized cell types has been applied for epilepsy treatment in preclinical animal research and clinical trials. In this review, we focused on the advances in stem cell therapy for epilepsies. The goals of stem cell transplantation, its mechanisms underlying graft effects, the types of grafts, and their therapeutic effects were discussed. The cell and animal models used for investigating stem cell technology in epilepsy treatment were summarized.

## Introduction

Epilepsy is a spectrum of heterogenous chronic brain disorders characterized by an enduring propensity for the generation of seizures which are the recurrent abnormal and hypersynchronous firing of neurons from dysfunctional brain circuits. Epilepsy remains one of the most prevalent neurological conditions affecting approximately 50–60 million people worldwide (WHO, 2022). Epilepsy can be classified into three major categories based on the possible underlying etiologies: idiopathic (genetic), symptomatic (acquired) including structural, infectious, metabolic, and immune etiologies, as well as unknown/cryptogenic (presume symptomatically) (Engel, 2011; Scheffer et al., 2017). Pharmacological therapy is the mainstay of treatment for epilepsy and many new anti-seizure medications (ASMs) have been developed in the past decades (Galanopoulou et al., 2012; Loscher, 2017). However, nearly one-third of epilepsy patients still fail to achieve seizure control or soon become resistant to currently available therapies (Kwan and Brodie, 2000, 2004; Shorvon, 2009a,b). Presently available medications are based on symptomatic strategies aiming to suppress or reduce the frequency and severity of seizures. These approaches do not affect and modify the underlying processes of epilepsy (Galanopoulou et al., 2012; Loscher et al., 2013). There is an unmet need to develop a therapeutic strategy for epilepsy that more specifically targets dysfunctional epileptic circuitry, particularly for medically refractory epilepsy and for suppressing the development and progression of epilepsy.

Stem cells are “undifferentiated” cells that have not yet engaged in a developmental path to form a specific cell type or tissue. These cells have the potential to be differentiated into a variety of specialized post-mitotic cell lineages (Kolios and Moodley, 2013). Stem cells can be classified into four major categories according to their origins: (1) Embryonic stem cells (ESCs), (2) Infant stem cells such as umbilical cord stem cells, (3) Adult stem cells (ASCs) including tissue-resident stem cells (e.g. ectoderm), neural stem cells, mesenchymal stem cells (MSCs) which can be obtained from bone marrow and adipose tissue, and (4) induced pluripotent stem cells (iPSCs) which are genetically reprogrammed adult stem cells to exhibit characteristics of ESCs (Fortier, 2005; Kolios and Moodley, 2013; EL Barky et al., 2017). These stem cells possess different differentiation potencies that are totipotent, pluripotent, multipotent, unipotent, and oligopotent cells (EL Barky et al., 2017). Totipotent cells have the capacity to differentiate into any cell type, form any tissue and produce an entire organism. Pluripotent cells have the potential to give rise to nearly all cells containing the three germ layers of the embryo, endoderm, mesoderm, and ectoderm. Multipotent cells can restrictedly differentiate to cell types related to their origin tissues. Unipotent cells and oligopotent cells can only be specialized in limited cell lineages of their own kind (EL Barky et al., 2017). As stem cells have the ability of self-replication and transform into specialized cell types, they play an important role in tissue repair and regeneration and provide the potential to restore and integrate the dysfunctional brain circuits to a normal state.

The therapeutic use of various stem cell types for the treatment of epilepsy has been increasingly explored in experimental and clinical research (Table 1). The pathophysiological mechanisms of epilepsy are attributed to a dysfunctional receptor or molecule expression of neural cells within the brain, neuronal damage or loss, astrocyte activation, reactive oxygen species production, and mitochondrial dysfunction. Stem cell-based treatment provides an attractive approach for replacement of the damaged or lost cells, correcting the imbalance of excitatory and inhibitory electrical activity in epileptic focus and brain circuits. Growing studies show that stem cell transplantation therapy is a novel promising therapeutic strategy for epilepsy. In this review, we focus on recent advancements in stem cell therapy for epilepsies and discuss the potential mechanisms, various graft sources, and their effects. We also review the disease models used for studying stem cell technology in epilepsy treatment.

**Table 1:** Preclinical and clinical studies of stem cell therapy for epilepsy.

**Type of stem cells**	**Animal model or clinical trail**	**Route of administration**	**Conclusion**	**References**
Rat ADK-deficient ESC-derived NPs	Electrically-induced status epilepticus rat model	Intracerebral grafting into hippocampi	Retardation of epileptogenesis and prevented the occurrence of generalized seizures	Li et al., 2007
ESCs-derived EMG	Transgenic rodent model	Intracerebral injection into cortex	Reduction of the duration and frequency of spontaneous electrographic seizures	Baraban et al., 2009; Hammad et al., 2015
ESCs-derived EMG	Rodent model	Intracerebral grafting into hippocampi	Reduced electrographic seizures and improved spatial learning, hyperactivity and aggressive response	Hunt et al., 2013; Casalia et al., 2017
hESCs	Rodent model	Intracerebral grafting into hippocampi	Decreased the frequency and duration of spontaneous recurrent seizures	Waloschkova et al., 2021
NSCs	Rodent model	Intravenous infusion	Histopathological analysis displayed alleviation of oxidative damage	de Gois da Silva et al., 2018
NSCs	Rodent model	Intracerebral grafting into hippocampi	Decreased the frequency and duration of spontaneous recurrent seizures	Xu et al., 2019; Lentini et al., 2021
NSCs	Rodent model	Intracerebral grafting into hippocampi	Restrained epileptogenesis, and decreased the frequency and duration of spontaneous recurrent seizures. Reduced cognitive and mood impairments	Hattiangady et al., 2020
MSCs	Rodent model	Intravenous infusion	Mitigated epileptogenesis and preserved cognitive function	Fukumura et al., 2018; Ali et al., 2019
Adipose-derived MSCs	Rodent model	Intracerebral transplanting into hippocampus	Alleviated EEG burst and improved learning and memory function	Wang et al., 2021
Autologous bone marrow mononuclear-derived MSCs	Clinical trial	Intravenous and intrathecal injection	Safe and significantly reduced seizure frequency, and have unique immunomodulatory properties	Hlebokazov et al., 2017, 2021
Autologous bone marrow- derived MSCs	Clinical trial	Intravenous and intrathecal injection	Mitigated seizure frequency, improved cognitive function and no adverse events.	Milczarek et al., 2018
Autologous bone marrow- derived MSCs	Clinical trial	Intra-arterial infusion	40% patient seizure free at six months follow up, and significant memory improvement	DaCosta et al., 2018
Autologous bone marrow- derived MSCs	Clinical trial	Intravenous injection	Remission of seizure activity in 65% of patients	Hammadi, 2019
Autologous adipose-derived MSCs	Clinical trail	Intrathecal infusion	Seizure free in only 1 of 6 patients, and unsatisfactory seizure reduction or no apparent remission in others	Szczepanik et al., 2020
hiPSCs	Rodent model	Intracerebral grafting into hippocampi	Reduced seizure activity and ameliorated memory impairment, hyperactivity and aggressive behaviors	Cunningham et al., 2014; Upadhya et al., 2019

*ADK, adenosine kinase; ESCs, Embryonic stem cells; hESCs, human embryonic stem cells; NSCs, Neural stem cells; NPs, Neuronal precursor cells; MSCs, Mesenchymal stem cells; hiPSCs, human induced pluripotent stem cells*.

## Mechanisms of Action of Neurotransplantation for Epilepsy

The fundamental pathophysiologic basis of epilepsy is the transformation of the brain from a normal neuronal network to a long-lasting hyperexcitable state owing to an imbalance between the excitatory neurons and inhibitory neurons in the brain. Theoretically, stem cell transplantation could have disease-modifying effects to restore the malfunction of the epileptic brain *via* diverse mechanisms including specific cell substitution, rescue and repair of degeneration cells, reorganization of synapses, modulation of the secretion of neurotransmitters or beneficial neurotrophic factors (Li et al., 2007; Roper and Steindler, 2013; Hattiangady et al., 2020).

### Cell Substitution

Epilepsy is often associated with either programmed or unprogrammed cell death. Replacement of damaged and lost cells or specific cell lines such as implantation of GABAergic interneurons integrating into pre-existing brain circuitry to achieve the suppression of hyperexcitation of neural networks (Waldau et al., 2010; Shetty and Upadhya, 2016).

### Cell Rescue

The regenerative properties of grafted stem cells are capable to ameliorate host cells from the degenerative processes, repair myelin sheaths of axons in the impaired neurons and enhance neurogenesis, and restrain anomalous mossy fiber sprouting in the epileptic brain (Uchida et al., 2012; Kodali et al., 2019; Hattiangady et al., 2020). Stem cells also exhibit neuroprotective capacity by attenuating oxidative stress and glutamate excitotoxicity associated with epilepsy (de Gois da Silva et al., 2018; Papazian et al., 2018; Luo et al., 2019).

### Supplement of Beneficial Neurotrophic Factors

Grafted stem cells can differentiate into glial cells which have an important role in secreting many neurotrophic factors and molecules including brain-derived neurotrophic factor (BDNF), glial-derived neurotrophic factor (GDNF), insulin-like growth factor-1 (IGF-1) and microRNA to support neuron survival, neurogenesis and maintain normal neuronal function (Waldau et al., 2010; Lai et al., 2014; Ali et al., 2019; Hattiangady et al., 2020; Wang et al., 2021).

### Anti-Inflammatory Properties

MSCs and NSCs could suppress the neuroinflammation process via inhibiting the secretion of proinflammatory factors and promoting the release of anti-inflammatory factors, as well as modulate neuroimmune responses through direct cell contact (Ryu et al., 2009; Cao et al., 2011; Wang et al., 2014; Kim et al., 2020).

## Therapeutic Applications and Effects of Stem Cells in Epilepsy

The utility of *in vitro* stem cell technology by patient-derived iPSCs to high-throughput screening of anti-seizure medications can be applied for personalized epilepsy therapy (Jiao et al., 2013; Kaufmann et al., 2015; Elitt et al., 2018; Lybrand et al., 2020). The potential major roles of stem cell therapy in epilepsy are seizure remission, inhibition of the progression of the epileptogenic process, prophylaxis against the development of chronic epilepsy, and improvement of cognitive function (Aligholi et al., 2021; Sadanandan et al., 2021; Ramos-Fresnedo et al., 2022). A phase I clinical trial study showed a favorable seizure reduction, a good safety profile, and improvement of pathological hallmarks in epilepsy patients treated with autologous MSCs (Hlebokazov et al., 2017).

## Sources and Types of Stem Cells

The pathogenesis of epilepsy is not only limited to dysfunction of neurons (such as glutamatergic and GABAergic cells) but also involves glia such as astrocytes and microglia. Various sources and many types of cells have been tested as a novel treatment in epilepsy to explore the therapeutic potency and safety of stem cell therapy, particularly for drug-resistant epilepsy.

### Embryonic Stem Cells (ESCs)

Embryonic stem cells are pluripotent that can be differentiated into almost any cell lineage. Cortical inhibitory interneurons mainly originate from the embryonic medial and caudal ganglionic eminences in the forebrain during development (Wichterle et al., 1999; Xu et al., 2004; Butt et al., 2005). Grafting medial ganglionic eminence (MEG) progenitors into the brain exhibits normal migratory activity and can functionally differentiate as GABAergic inhibitory neurons and integrate into the neural circuitry to suppress seizures (Baraban et al., 2009; Hunt et al., 2013; Casalia et al., 2017). However, transplantation of rodent embryonic caudal ganglionic eminence (CGE) was not shown a reduction in seizure activity (Casalia et al., 2017). Despite the embryonic stem cells owning a great differentiation capacity to various cell types, the ethical concerns limit the utility of embryonic stem cells harvested from humans for stem cells.

### Neural Stem Cells (NSCs)

Neural stem cells are multipotent cells capable of self-renewing and generating three major cell types of the central nervous system: neurons, astrocytes, and oligodendrocytes (Al-Mayyahi et al., 2018). In the adult brain, only two regions have the ability of neurogenesis: the subventricular zone of the lateral ventricle and the subgranular zone of the hippocampal dentate gyrus (Parent et al., 1997; Miltiadous et al., 2013). NSCs can be harvested from several sources such as fetal, postnatal, and adult brains, and also can be generated from ESCs and iPSCs (Hattiangady and Shetty, 2011; Noebels et al., 2012). In the animal model of temporal lobe epilepsy, transplanting NSCs into the hippocampus appears responsive effects on seizure remission and has the potential to mitigate and repair epilepsy-induced pathological changes (Hattiangady and Shetty, 2012; Noebels et al., 2012; Uchida et al., 2012; de Gois da Silva et al., 2018; Xu et al., 2019). While NSCs show promising potential for treating mesial temporal lobe epilepsy, clinical research is paucity and a lack of data to demonstrate the effectiveness of NSC therapy on other forms of epilepsy.

### Mesenchymal Stem Cells (MSCs)

Mesenchymal stem cells are multipotent cells that can be isolated from bone marrow, adipose tissue, umbilical cord blood, placenta, and amniotic fluid (Kolios and Moodley, 2013; Berebichez-Fridman and Montero-Olvera, 2018). MSCs possess self-renewal capacities and are able to differentiate into many specialized cell types including neurons (Tropel et al., 2006). Recently, several preclinical animal and clinical studies have investigated the applications of MSCs for the treatment of epilepsy and appear potentials to ameliorate the severity of epilepsy and attenuate epilepsy-induced excitotoxicity, oxidative stress, and neuroinflammation (Fukumura et al., 2018; Milczarek et al., 2018; Papazian et al., 2018; Kodali et al., 2019; Xian et al., 2019; Linares et al., 2020). Furthermore, stem cell therapy using MSCs allows either allogeneic or autologous transplantation that extends the clinical feasibility. Clinical trials with autologous MSCs obtained from human bone marrow or adipose-derived regenerative cells for the treatment of medically refractory epilepsy display variable efficacy of seizure reduction with the improvement of cognitive and psychomotor function and good safety of the therapy (Hlebokazov et al., 2017; Milczarek et al., 2018; Szczepanik et al., 2020).

### Induced Pluripotent Stem Cells (iPSCs)

Induced pluripotent stem cells can be non-invasively derived from adult somatic cells such as fibroblast or peripheral mononuclear blood cells undergoing genetic reprogramming to have pluripotent properties (Yu et al., 2007; Braganca et al., 2019). iPSCs are able to differentiate into many neural cell types including distinct subtypes of GABAergic interneurons, astrocytes, motor neurons, and Purkinje cells (Du and Parent, 2015; Wang et al., 2015; Watson et al., 2018). Specific cell types derived from iPSCs implanting into the epileptic brain can migrate extensively into different subfields of the target brain region with synaptic integration of grafted cells into the dysfunctional circuitry (Cunningham et al., 2014). Grafting maturing GABAergic interneurons derived from human-induced pluripotent stem cells (hiPSCs) in an epilepsy mouse model has been shown to mitigate seizures and improve cognitive function, aggressive behavior, and mood (Cunningham et al., 2014). Implanting hiPSCs-derived MGE-like interneuron precursors into epileptic rodent hippocampi showed anti-epileptic and anti-epileptogenic effects with significantly restraining seizure phenotypes and alleviating cell loss, abnormal neurogenesis, and aberrant mossy fiber sprouting (Noakes et al., 2019; Upadhya et al., 2019). Nonetheless, there is still a lack of clinical data showing the application of iPSCs for the treatment of human epilepsy.

## Disease Models and Animal Model Studies

### Use of iPSCs to Model Diseases

Patient-derived iPSCs are models of a patient's genetic background and can be used as a platform for modeling patient-specific diseases, human genetic epilepsies, high throughput drug screening, and studying the mechanisms of epilepsy (Sun et al., 2016; Elitt et al., 2018; Kim et al., 2018).

### Stem Cell Transplants in Animal Models of Epilepsy

Mesial temporal lobe epilepsy (mTLE) which mainly involves the hippocampus is often medically refractory. Animal models mimic mesial temporal lobe epilepsy including kainic acid and pilocarpine rodent models are commonly used in chronic epilepsy models for stem cell research (Cunningham et al., 2014; Casalia et al., 2017; Upadhya et al., 2019; Lentini et al., 2021; Waloschkova et al., 2021). Electrically-induced status epilepticus model is another applicable rodent model of status epilepticus and chronic mTLE (Li et al., 2007). Genetic or transgenic mice such as Kv1.1 mutation which associates with human episodic ataxia type 1 and focal cortical epilepsy, and Stargazer (stg) transgenic mouse model of absence epilepsy have been applied to explore the therapeutic effects on epilepsy by transplantation of GABAergic interneurons into the brain (Baraban et al., 2009; Hammad et al., 2015). Pentylenetetrazole and picrotoxin rodent models are acute seizure models that can be used to evaluate the anticonvulsant effects of cell-based therapy (Handreck et al., 2014; de Gois da Silva et al., 2018).

## Perspectives of Clinical Application of Neurotransplantation of iPSCs for Epilepsy

iPSCs obtained from patients themselves can be manipulated or corrected by novel genetic editing technology such as CRISPR/Cas9, then autologously implanted into patients to repair their underlying pathological circuits in the epileptic brain (Hockemeyer and Jaenisch, 2016; Dever et al., 2019). Combining the iPSCs and genetic engineering technology might serve as a potential disease-modifying treatment for epilepsy and personalized therapy.

## Conclusions

Emerging evidence suggests that stem cell approaches could be a promising tool in epilepsy treatment. However, further studies are required to investigate and address the optimal regimen, microenvironment, and timing of stem cell differentiation and implantation. In addition, compared to ESCs and NSCs, MSCs and iPSCs do not raise ethical issues. Autologous cell transplants avoid the immune response and have better integration between the graft and host cells which would be a good option for the development of cell-based therapy. Advanced preclinical investigations in clinically relevant models of epilepsy and larger clinical trials need to be conducted to further demonstrate the efficacy and safety of stem cell therapy for epilepsy.

## Author Contributions

B-LC: conceptualization and funding acquisition. B-LC and K-HC: writing—original draft and writing—review and editing. Both authors contributed to the article and approved the submitted version.

## Funding

This work was supported by grants from Chang Gung Memorial Hospital, Taipei, Taiwan (CMRPG3I0521, CMRPG3K1021, CMRPG3L0661, and BMRPJ26) and the Ministry of Science and Technology, Taiwan (MOST 108-2314-B-182A-153 -, MOST 109-2314-B-182-079-, and MOST 110-2314-B-182-055-).

## Conflict of Interest

The authors declare that the research was conducted in the absence of any commercial or financial relationships that could be construed as a potential conflict of interest.

## Publisher's Note

All claims expressed in this article are solely those of the authors and do not necessarily represent those of their affiliated organizations, or those of the publisher, the editors and the reviewers. Any product that may be evaluated in this article, or claim that may be made by its manufacturer, is not guaranteed or endorsed by the publisher.
